# Complete Genome Sequence of Bacillus amyloliquefaciens Strain BH072, Isolated from Honey

**DOI:** 10.1128/genomeA.00098-15

**Published:** 2015-03-12

**Authors:** Xin Zhao, Anne de Jong, Zhijiang Zhou, Oscar P. Kuipers

**Affiliations:** a Molecular Genetics, Groningen Biomolecular Sciences and Biotechnology Institute, University of Groningen, Groningen, The Netherlands; b School of Chemical Engineering and Technology, Tianjin University, Tianjin, China

## Abstract

The genome of Bacillus amyloliquefaciens strain BH072, isolated from a honey sample and showing strong antimicrobial activity against plant pathogens, is 4.07 Mb and harbors 3,785 coding sequences (CDS). Several gene clusters for nonribosomal synthesis of antimicrobial peptides and a complete gene cluster for biosynthesis of mersacidin were detected.

## GENOME ANNOUNCEMENT

Among biocontrol microorganisms, Bacillus amyloliquefaciens strains have the ability to enhance the yield of crop plants and to suppress microbial plant pathogens (1). Recently, B. amyloliquefaciens subsp. amyloliquefaciens strain DSM7 (2) and plant-associated Bacillus amyloliquefaciens subsp. plantarum group strains FZB42, YAU B9601-Y2, and CAU B946 (3–6) have been completely sequenced. All these strains contain 7 or more gene clusters for either ribosomally encoded bacteriocins or nonribosomal antimicrobial polyketides or lipopeptides. Here, we report the genome sequence of the B. amyloliquefaciens strain BH072 that also contains gene clusters similar to those of the 4 strains mentioned above, but in a different combination.

Strain BH072, a novel bacterium isolated from a honey sample, was identified as being B. amyloliquefaciens by 16S rRNA gene and *gyrA* gene sequencing (7) and physiological and biochemical analysis. It had a broad spectrum of antifungal activity against various molds, such as Aspergillus niger, *Pythium*, Fusarium oxysporum, and Botrytis cinerea. The *ituA*, *hag*, and *tasA* genes, encoding iturin A, flagellin, and TasA, were detected by PCR analysis, and flagellin and iturin A were purified and identified by Zhao et al. (8).

Genomic DNA prepared from strain BH072 was sequenced using Pacific Biosciences RS sequencing technology (Pacific Biosciences, Menlo Park, CA), yielding >100× average genome coverage. The sample was prepared as a 10-kb insert library and sequenced on a silencing mediator of retinoic acid and thyroid hormone receptor (SMRT) v.2.3 cell. The Hierarchical Genome Assembly Process (HGAP3) workflow (PacBio DevNet; Pacific Biosciences) was used to perform a *de novo* assembly. The genome was annotated at the National Center for Biotechnology Information (NCBI) using the Annotation pipeline 2.9 (rev. 452132).

The complete genome sequence of BH072 was composed of one circular contig of a 4,069,641-bp chromosome with a G+C value of 46.4%. The genome was larger than that of strains FZB42, CAU B946, and DSM7 but smaller than strain YAU B9601-Y2. The chromosome consisted of 3,943 genes, 3,785 CDSs, 44 pseudogenes, 27 rRNAs (5S, 16S, 23S), and 86 tRNAs. Phylogenetically the strain YAU B9601-Y2 is the closest neighbor of B. amyloliquefaciens strain BH072. Remarkably, based on BAGEL3 mining (9), the whole mersacidin operon was detected in BH072, making it the third mersacidin producer strain until now, the other two being YAU B9601-Y2 and HIL Y-85 (6, 10).

### Nucleotide sequence accession number.

The genome sequence of B. amyloliquefaciens BH072 has been deposited in Genbank, under the accession number CP009938.
